# LINC‐PINT alleviates lung cancer progression via sponging miR‐543 and inducing PTEN

**DOI:** 10.1002/cam4.2822

**Published:** 2020-01-25

**Authors:** Shu Wang, Wenyang Jiang, Xinghua Zhang, Zilong Lu, Qing Geng, Wei Wang, Nan Li, Xinyong Cai

**Affiliations:** ^1^ Department of Gerontology The First Affiliated Hospital of Nanchang University Nanchang China; ^2^ Department of Thoracic Surgery Renmin Hospital of Wuhan University Wuhan China; ^3^ Department of Cardiology Shangrao People's Hospital Shangrao China; ^4^ Department of Cardiology Jiangxi Provincial People's Hospital Affiliated to Nanchang University Nanchang China

**Keywords:** lncRNA, lung cancer, miR‐543, PINT, PTEN

## Abstract

Long intergenic nonprotein coding RNA p53‐induced transcript (LINC‐PINT) has been reported to participate in various cancers. Here, we investigated the effects of LINC‐PINT on lung cancer progression. Firstly, in our study, we implied that LINC‐PINT was obviously decreased in NSCLC. Thereafter, in A549 and H1299 cells, LINC‐PINT was upregulated via transfecting LV‐LINC‐PINT. As exhibited, LINC‐PINT repressed cell proliferation and cell colony formation of A549 and H1299 cells. Subsequently, flow cytometry evidenced that A549 and H1299 cell apoptosis was obviously triggered and the cell cycle was arrested in G1 phase. Then, migration and transwell invasion experiments were carried out to detect the cell migration and invasion capacity. We found A549 and H1299 cell migration and invasion capacity were restrained by the upregulation of LINC‐PINT. Meanwhile, we predicted that miR‐543 could function as the target of LINC‐PINT and the association was verified. Moreover, we exhibited that miR‐543 was remarkably increased in lung cancer, which could be regulated by LINC‐PINT negatively. Furthermore, PTEN could act as the downstream target of miR‐543 and upregulation of miR‐543 repressed PTEN, which was reversed by LV‐PINT in A549 and H1299 cells. Finally, xenografts were utilized to confirm the function of LINC‐PINT on lung cancer. All these findings concluded that LINC‐PINT exerted crucial biological roles in NSCLC through sponging miR‐543 and inducing PTEN.

## Graphical abstract

LINC‐PINT was greatly reduced whereas miR‐543 was up‐regulated in lung cancer. LINC‐PINT affected proliferation, metastasis and invasion in lung cancer via regulating miR‐543 and inducing PTEN.

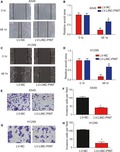

## 1 INTRODUCTION

Lung cancer is still a serious public health problem worldwide. In these ten years, The incidence of lung cancer is still increasing a lot with about 1.6 million patients diagnosed every year.^1^, ^2^ NSCLC can account for about 80% of lung cancer.^3^ Recently, great advancements have been made in NSCLC. Nevertheless, its prognosis is still poor.^4^ Therefore, identifying the effective biomarkers of lung cancer is of great significance to improve the overall prognosis.

LncRNAs are crucial regulators of various diseases including cancers.^5^ LncRNAs are long noncoding RNA with over 200nts without protein‐coding functions.^6^ Recently, altered lncRNAs are recognized in various cancers.^7^, ^8^, ^9^ For example, lncRNAMEG3 can repress gastric cancer proliferation through regulating p53.^10^ LncRNACPS1‐IT1 can inhibit colorectal cancer cell invasion and metastasis.^11^ Upregulated lncRNA SNHG1 can contribute to lung cancer development through sponging miR‐101‐3p and activating Wnt signaling.^12^ LncRNA‐HIT promotes NSCLC progression by regulating ZEB1.^13^

In recent years, LINC‐PINT presents tumor inhibitory potential in many cancers.^14^, ^15^ For instance, LINC‐PINT can inhibit pancreatic ductal adenocarcinoma by activating TGF‐β signaling.^16^ LINC‐PINT can activate the MAPK signaling to induce acute myocardial infarction via sponging miR‐208a‐3p.^17^ However, whether LINC‐PINT affects the biological behavior of NSCLC cells through regulating miRNAs remains undetermined. Hence, in this research, we focused on exploring the interaction between LINC‐PINT and miRNAs in lung cancer cells. We reported that a novel pathway of PINT/miR‐543/PTEN is involved in the progression of NSCLC. Our study implied that LINC‐PINT could function as a potential biomarker and therapeutic target for lung cancer diagnosis and therapy.

## 2 MATERIALS AND METHODS

### 2.1 Clinical samples

Twenty paired tumor tissues and matched noncancerous tissues were collected in our hospital. Before the enrollment, all the patients signed the written informed consent and this study was approved by the Ethics Committee of Renmin Hospital of Wuhan University.

### 2.2 Cell culture

A549, H460, H1650, H1299, WI‐38, and HEL‐1 were obtained from the Cell Bank of the Chinese Academy of Science. Then, RPMI 1640 medium (Gibco) added with 10% FBS (Gibco) was used. A humidified incubator at 37°C with 5% CO_2_ was employed to maintain the cells.

### 2.3 Cell transfection

Both A549 and H1975 cells were grown in complete medium for a whole night to reach 60%‐70% confluence; miR‐543 mimics and the NCs were designed by GenePharma. Complete length cDNA of LINC‐PINT was amplified from lung cancer cells. Objective products were cloned into pcDNA3.1 with lentivirus packaging vectors pMD2.G. Medium with 800 μg/mL G418 was employed to incubate the cells. Lipofectamine 3000 reagent (Invitrogen) was utilized. Then, after 6‐8 hours of transfection, we replaced the medium with fresh complete medium.

### 2.4 Cell proliferation assay

After transfection for 48 hours, cells were harvested and seeded into a 96‐well plate. Afterward, the adhered cells were incubated with CCK‐8 solution (Beyotime Institute of Biotechnology) in the dark for 2 hours. The absorbance at 450 nm was detected using a microplate reader (Bio Tek Instruments).

### 2.5 5‐ethynyl‐20‐deoxyuridine (Edu) proliferation assay

EdU Cell Proliferation Assay Kit (C10310, Ribobio) was conducted; 50 µmol/L EdU was used for 4 hours. 1 µg/mL DAPI was used to stain the cell nuclei for 20 minutes. Fluorescence microscopy was employed.

### 2.6 Colony formation

To carry out the colony formation assay, 2000 cells were placed in a 6‐well plate and cultured in the medium containing 10% FBS for 2 weeks. Colonies were fixed with methanol and stained with 0.1% crystal violet (1 mg/mL). Cell colonies were stained using Coomassie brilliant blue (Beyotime). A fluorescence microscope (IX70, Olympus) was utilized to count the cell colonies.

### 2.7 Analysis of cell cycle

Cells were fixed by 70% ethanol for a whole night. Thereafter, cells were added to 1 mg/mL RNase A and 0.1 mg/mL PI in the dark for half an hour. Distribution of cell cycle was evaluated by a FACScan flow cytometer (BD Biosciences). FlowJo was utilized to investigate the ratios of cells.

### 2.8 Analysis of cell apoptosis

An Annexin V‐FITC/PI detection kit (BD Pharmingen Poland, 556 547) was used to measure apoptosis of A549 and H1975 cells. After transfection, cells were centrifuged at 2000 rpm for 5 minutes. Precold 1 PBS was used to suspend cells and then centrifuged again at 200 rpm for 10 minutes. Next, cells were stained by Annexin V‐FITC and PI for 15 minutes in avoidance of light. Then, fluorescence intensities were determined using the Partec Cube 6 flow cytometer (Partec).

### 2.9 Scratch assay

For starvation, serum‐free medium was employed to maintain the cells. A P200 pipette tip was used to scratch the monolayer. In order to observe the wound areas, an inverted microscope was used.

### 2.10 Transwell invasion assay

The upper chambers (Cat. No, 3422 CORSTOR) were coated by Matrigel for 2 hours before growing the cells. Cells were harvested and then they were plated into the upper chambers. A quantity of 750 μL 10% FCS media was loaded to the lower chambers. The invaded cells were permeabilized using methanol for 20 minutes and in order to stain the cells, crystal violet was employed in a dark room.

### 2.11 QRT‐PCR

The total RNA was isolated by TRIzol reagent (cat. no. 9109, TaKaRa). Afterward, cDNA was prepared via reverse transcription of RNA based on the instructions of Prime Script RT Reagent Kit (RR037A, TaKaRa). Here, qRT‐PCR reaction was performed using SYBR Premix Ex Taq II (RR820A, TaKaRa). Applied Biosystems 7900 Real‐Time PCR System was employed and the 2^−ΔΔCt^ method was used. The primers used in this study are listed in Table 1. GAPDH and U6 were employed as internal references.

**Table 1:** Primers for real‐time PCR

Genes	Forward (5ʹ‐3ʹ)	Reverse (5ʹ‐3ʹ)
GAPDH	TATCGGACGCCTGGTTAC	TATCGGACGCCTGGTTAC
PINT	CGTGGGAGCCCCTTTAAGTT	GGGAGGTGGCGTAGTTTCTC
miR‐543	CCAGCTACACTGGGCAGCAGCAATTCATGTTT	CTCAACTGGTGTCGTGGA
U6	CTCGCTTCGGCAGCACATATACT	ACGCTTCACGAATTTGCGTGTC
PTEN	GAGCGTGCAGATAATGACAAGGAAT	GGATTTGACGGCTCCTCTACTGTTT

### 2.12 Western blot

Total proteins were extracted from using radioimmuno precipitation assay lysis buffer (Beyotime). Proteins were separated using PAGE with a 5% stacking gel and a 10% separating gel and then the proteins were transferred onto a PVDF membrane (cat. no. IPVH00010). Thereafter, the membrane was blocked in 5% skimmed milk. Rabbit anti‐human antibody of PTEN (cat. no 9188, 1:1000, CST) and GAPDH (cat. no 5174, 1:1000, CST) were utilized to incubate the membrane at 4°C. Secondary antibody (1:2000, CST) was added to the membrane. Enhanced chemiluminescence (ECL) solution (cat. no. WBKLS0500, Millipore) was employed to expose the protein bands. Protein bands were visualized in a gel imaging system (MG8600, Bio‐Rad). Images were analyzed by the Image‐Pro Plus software (Version 7.0, Media Cybernetics).

### 2.13 Dual‐luciferase reporter assay

The sequence of PINT and the 3ʹUTR of PTEN were cloned into the pmirGLO vectors (E1330, Promega) to create WT‐PINT and WT‐PTEN‐WT plasmids. In addition, region‐specific mutagenesis was carried out to create MUT‐PINT and MUT‐PTEN plasmids. The pRL‐TK renilla luciferase reporter vector (E2241, Promega) acted as the internal reference. NC or miR‐543 mimic were co‐transfected with the constructed luciferase reporter vectors into A549 cells. The luciferase signals were measured by the dual‐luciferase reporter gene assay kit (GM‐040502A, Qcbio Science and Technologies).

### 2.14 RIP experiment

EZ‐Magna RIP RNA‐binding protein immunoprecipitation kit (Millipore Corp.) was employed to carry out the RIP assay. A549 cells were lysed using the lysis buffer added with RNasin (Takara Bio, Otsu) and protease inhibitor (Roche). The lysate was centrifuged for half an hour. The supernatant was subjected to immunoprecipitation with anti‐Ago2 or anti‐IgG‐coated magnetic beads. After incubation for 4 hours, the beads were washed three times using the wash buffer.

### 2.15 RNA pull‐down assay

RNAs were labeled by biotin using the Pierce RNA 3ʹEnd Desthiobiotinylation Kit (Thermo Fisher Scientific). Briefly, A549 cells were treated using 50 nmol/L biotinylated WT miR‐543 (WT‐bio‐miR‐543) or MUT miR‐543 (MUT‐bio‐miR‐543). After 48 hours, the cells were lysed using specific lysis buffer (Ambion) for half an hour. Thereafter, the lysate was incubated with streptavidin magnetic beads, precoated using RNase‐free BSA and yeast tRNA at 4C for 3 hours. The beads were then washed using the following buffer: precold lysis buffer (two times), low‐salt buffer (three times), and high‐salt buffer. qRT‐PCR was carried out to analyze LINC‐PINT content.

### 2.16 Xenograft tumors

Twelve BALB/c nude mice (Slac Laboratory Animal) were grouped as LV‐NC and LV‐LINC‐PINT. Mice were injected with A549 cells infected with LV‐NC or LV‐PINT intraperitoneally. The mice were sacrificed after 30 days. Thereafter the tumors were harvested for future studies. This animal research was approved by the ethic committee of Renmin Hospital of Wuhan University and was based on the Guide for the Care and Use of Laboratory Animals of the NIH.

### 2.17 HE staining

The histopathology of tumor tissues was carried out using hematoxylin‐eosin (HE) staining. Tissue samples was put into 10% formaldehyde solution and then dehydrated, embedded in paraffin. We cut down the tissues into slices of 4 μm. Afterward, the samples were stained by hematoxylin and eosin. Finally, the slices were observed under a light microscope (Leica Microsystems).

### 2.18 IHC staining

Briefly, sections were deparaffinized and rehydrated. Thereafter, antigen retrieval was carried out using Target Retrieval Solution. 0.3% hydrogen peroxide was used to block the endogenous peroxidase activity for 15 minutes. Slides were blocked using goat serum, avidin solution, and biotin solution. Slides were incubated with rabbit anti‐human polyclonal antibodies against Ki‐67 (1:500 dilution, cat. no 9449, 1:1000, CST) at 4°C for a whole night and then probed with biotinylated goat anti‐mouse secondary antibody (1:500 dilution, cat. no 8125, 1:1000, CST).

### 2.19 Statistical analysis

All data were analyzed using SPSS 22.0 statistical software (IBM). The data were exhibited as mean ± SD. An independent sample *t* test was used to analyze the differences between two groups. Comparisons among multiple groups were analyzed by one‐way ANOVA. Statistical significance was indicated with a *P *< .05.

## 3 RESULTS

### 3.1 LINC‐PINT was down‐regulated in lung cancer

**Figure 1:**
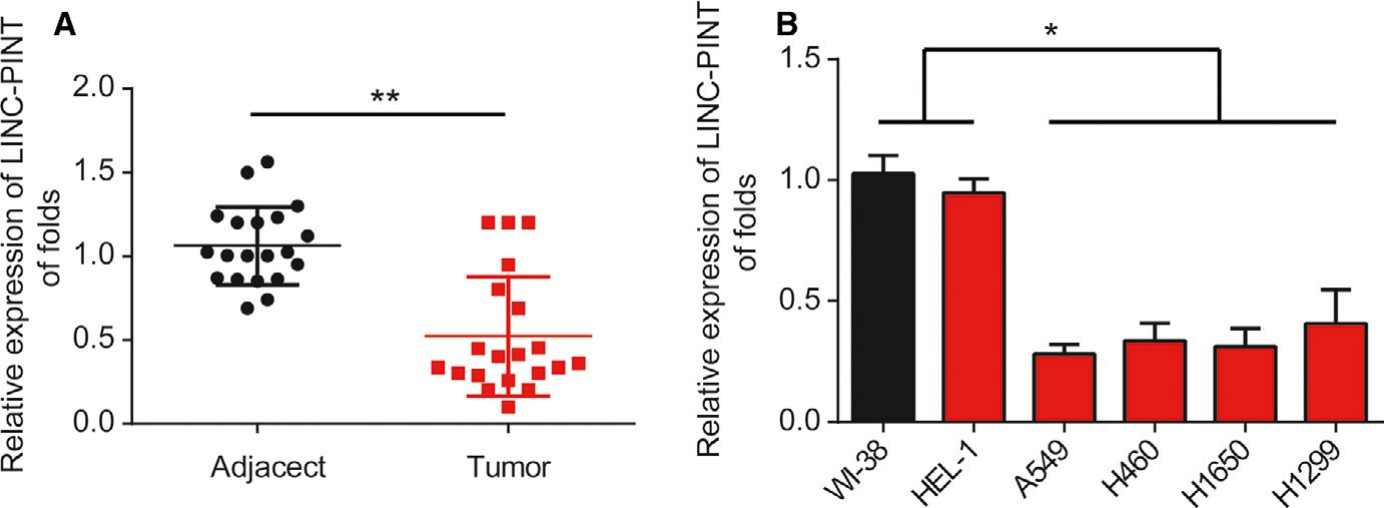
Expression of LINC‐PINT in NSCLC. A, LINC‐PINT expression in lung cancer tissues. B, LINC‐PINT expression in lung cancer cells (A549, H460, H1299, H1650) and WI‐38, HEL‐1 cells. LINC‐PINT expression was normalized against GAPDH expression. Three independent experiments were carried out. Error bars stand for the mean ± SD of at least triplicate experiments. *P < .05, **P < .01

Firstly, we tested expression level of LINC‐PINT in NSCLC using qRT‐PCR. We exhibited LINC‐PINT was greatly decreased in NSCLC tissues in comparison to the nontumor tissues (Figure 1A, *P* < .01). Association with LINC‐PINT and the clinicopathological characteristics of lung cancer patients were displayed in Table 2. In addition, the reduced expression of LINC‐PINT was also observed in lung cancer cells, including A549, H460, H1299, and H1650 cells compared to human lung cell lines WI‐38 and HEL‐1 (Figure 1B, *F* value = 51.01, *P* < .01). These results suggested LINC‐PINT was involved in NSCLC.

**Table 2:** Association with LINC‐PINT and the clinicopathological characteristics of lung cancer patients

	Num	LINC‐PINT		*P* value
		Low (12)	High (8)	
Gender				.324
Male	11	7	4	
Female	9	5	4	
Age (years)				.438
≥60	9	6	3	
<60	11	8	3	
Tumor size				.232
≥4 cm	8	5	3	
<4 cm	12	8	4	
Differentiation				**.002** *
Well, moderate	9	4	5	
Poor	11	5	6	
TNM				**.030** *
I‐II	12	8	4	
III/IV	8	5	3	
Lymph metastasis				.561
No	6	4	2	
Yes	14	9	5	

* *P* < .05 (in bold).

### 3.2 LINC‐PINT inhibited lung cancer cell proliferation

**Figure 2:**
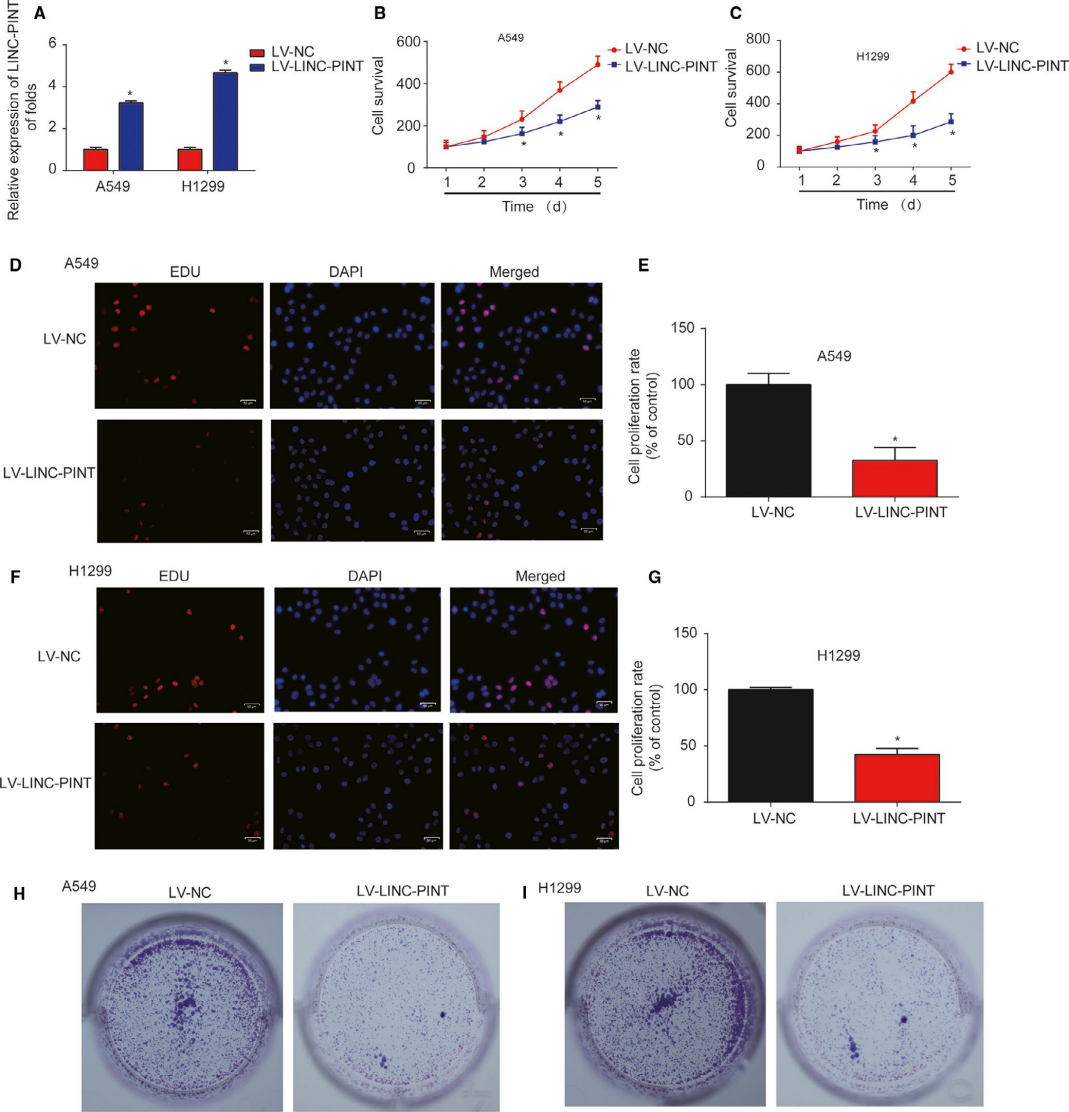
Effects of LINC‐PINT on NSCLC cell proliferation. A, LINC‐PINT expression in A549 and H1299 cells. Cells were infected with LV‐NC or LV‐LINC‐PINT for 48 h. B‐C, Effects of LINC‐PINT on the cell survival of A549 and H1299 cells. CCK8 assay was carried out to test cell viability. D‐E, Effects of LINC‐PINT on A549 cell proliferation. EdU assay was performed to detect cell proliferation. F‐G, Effects of LINC‐PINT on H1299 cell proliferation. H‐I, Effects of LINC‐PINT on the cell colony formation of A549 and H1299 cells. Colony formation assay was used to analyze the colony formation capacity. Three independent experiments were carried out. Error bars stand for the mean ± SD of at least triplicate experiments. *P < .05

Next, A549 and H1299 cells were overexpressed with LINC‐PINT by infecting LV‐LINC‐PINT. As exhibited in Figure 2A (*P* < .01), LINC‐PINT was greatly increased in A549 and H1299 cells. A549 and H1299 cell survival was repressed after infection of LV‐LINC‐PINT as proved by CCK‐8 assay (Figure 2B,C, *P* < .01). Consistently, EdU assay analysis indicated that overexpression of LINC‐PINT markedly inhibited lung cancer cell proliferation (Figure 2D‐G, *P* < .01). Additionally, colony formation assay indicated that A549 and H1299 cell colony formation capacity was greatly retrained by the upregulation of LINC‐PINT (Figure 2H,I, *P* < .01). These manifested that LINC‐PINT repressed lung cancer cell proliferation.

### 3.3 LINC‐PINT triggered lung cancer cell apoptosis and disturbed cell cycle

**Figure 3:**
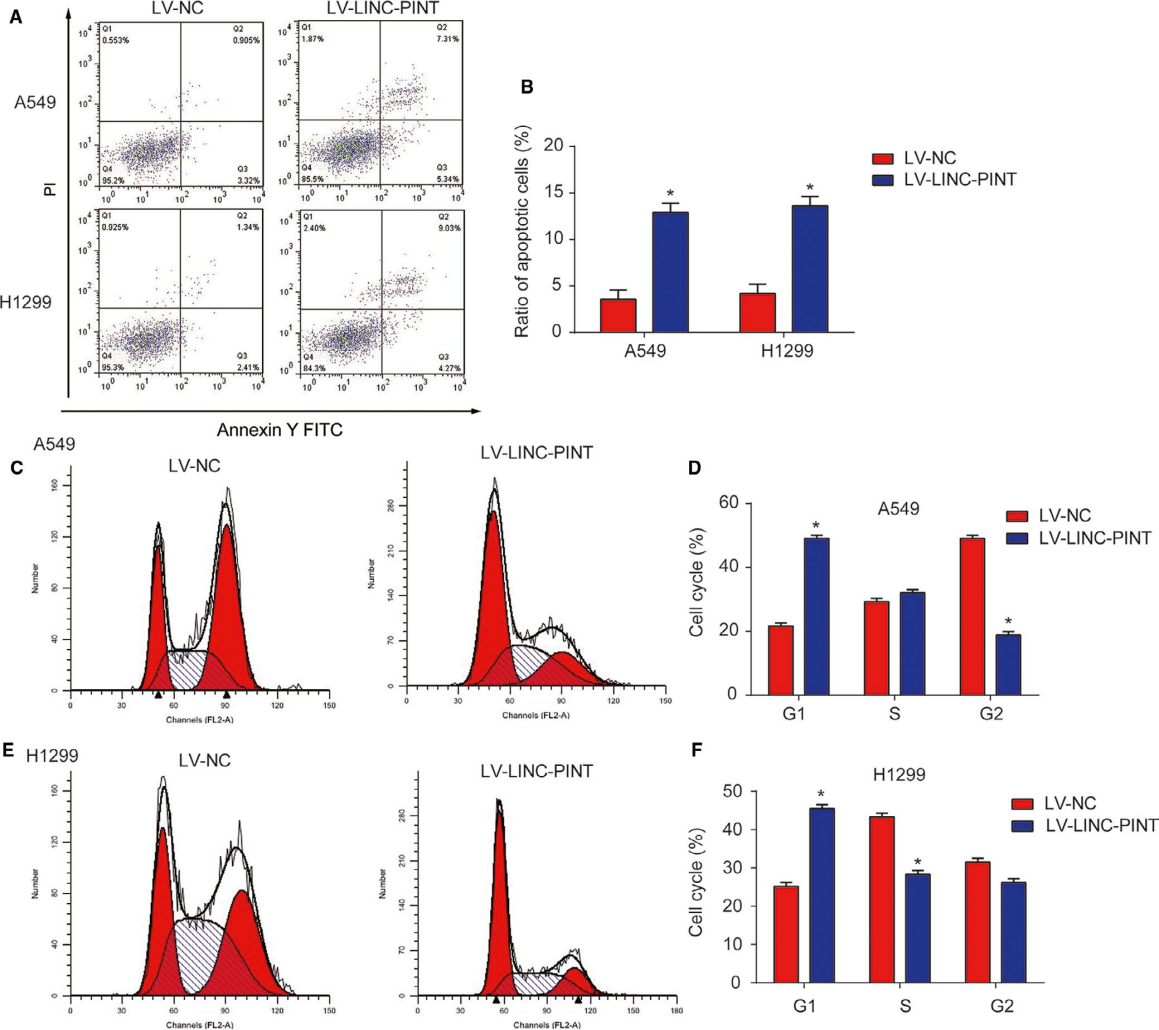
Effects of LINC‐PINT on lung cancer cell apoptosis and cell cycle. A‐B, Effects of LINC‐PINT on cell apoptosis of A549 and H1299 cells. Flow cytometry assay was used to analyze the cell apoptosis. C‐D, Effects of LINC‐PINT on the cell cycle in A549 cells. E‐F, Effects of LINC‐PINT on the cell cycle in H1299 cells. Three independent experiments were carried out. Error bars stand for the mean ± SD of at least triplicate experiments. *P < .05

In Figure 3A,B (*P* < .01), we found that A549 and H1299 cell apoptosis was obviously increased by overexpression of LINC‐PINT. In addition, as indicated in Figure 3C‐F (*P* < .01), lung cancer cell cycle was blocked in G1 phase by upregulation of LINC‐PINT in vitro. These results suggested that LINC‐PINT induced cell apoptosis and blocked cell cycle in lung cancer.

### 3.4 LINC‐PINT depressed cell migration and invasion ability

**Figure 4:**
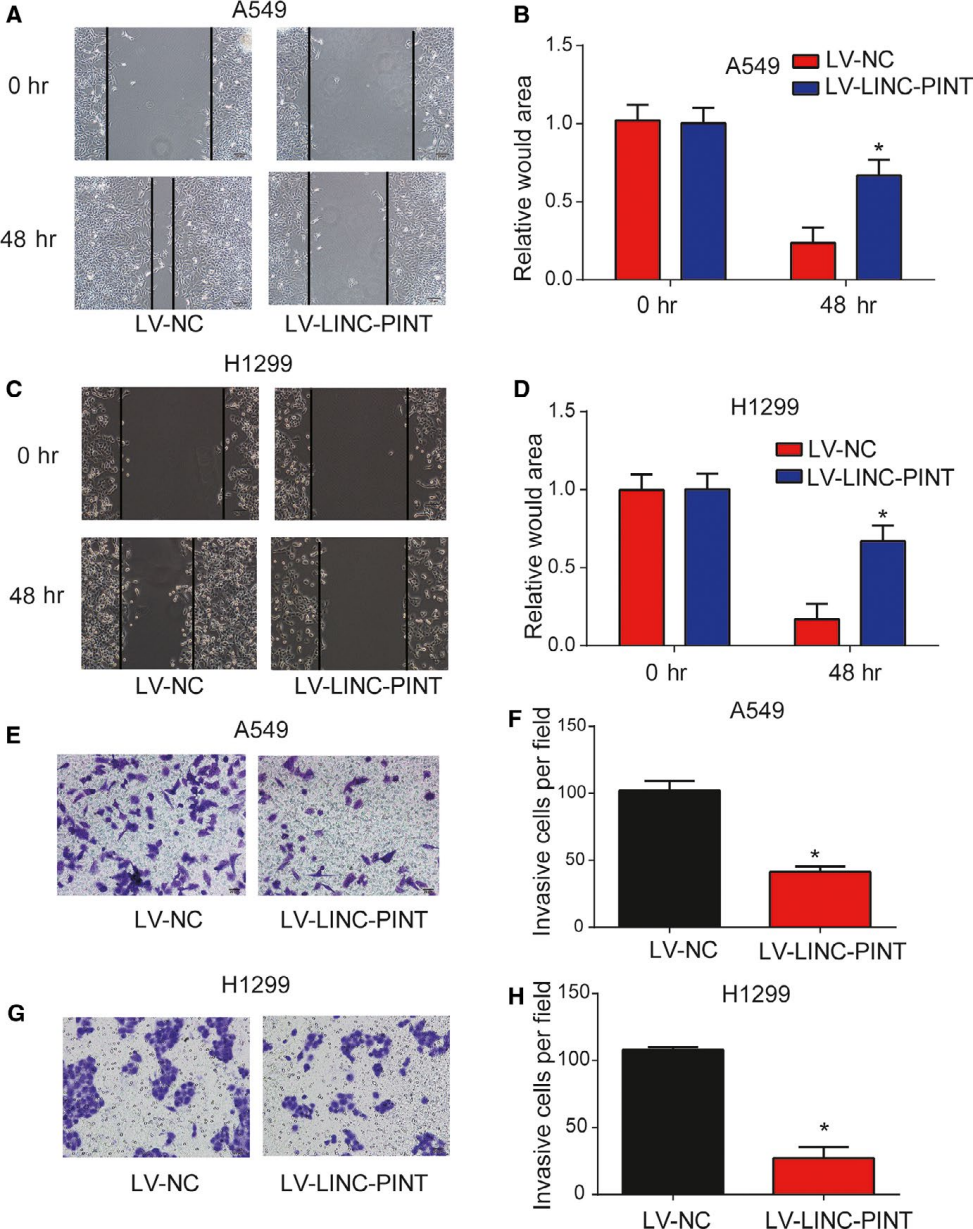
Effects of LINC‐PINT on lung cancer cell migration and invasion. A‐B, Effects of LINC‐PINT on the cell migratory ability of A549 cells. C‐D, Effects of LINC‐PINT on the cell migratory ability of H1299 cells. E‐F, Effects of LINC‐PINT on the cell invasion of A549 cells. G‐H, Effects of LINC‐PINT on the cell invasion of H1299 cells. Three independent experiments were carried out. Error bars stand for the mean ± SD of at least triplicate experiments. *P < .05

Subsequently, the migration capability of lung cancer cells was examined by wound‐healing assays. In Figure 4A‐D (*P* < .01), LINC‐PINT repressed the wound closure dramatically. Transwell invasion assay was carried out and in Figure 4E‐H (*P* < .01), increase in LINC‐PINT greatly retarded lung cancer cell invasion ability. These indicated LINC‐PINT depressed cell migration and invasion.

### 3.5 MIR‐543 was a direct target of LINC‐PINT

**Figure 5:**
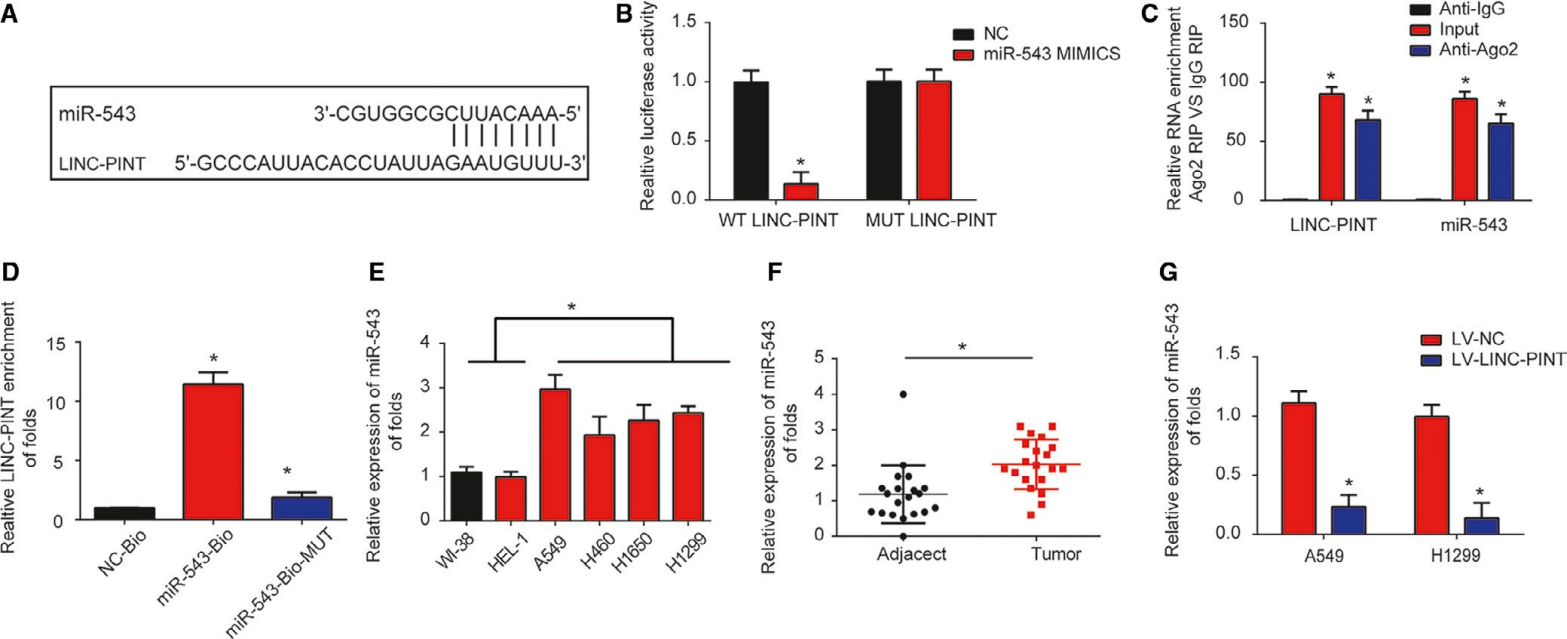
miR‐543 was a direct target of LINC‐PINT. A, Binding sites between LINC‐PINT and miR‐543. B, Luciferase activity was tested in A549 cells by transfected miR‐543 mimics, miR‐NC, WT‐LINC‐PINT, and MUT‐LINC‐PINT plasmid. C, The RIP assay indicated that both miR‐543 and LINC‐PINT were enriched in A549 cells. D, RNA pull‐down assay indicated the direct interaction between miR‐543 and LINC‐PINT. Cellular lysates were pulled down using biotinylated control (NC‐Bio), miR‐543 (miR‐543‐Bio), or miR‐543 probe containing mutations in the LINC‐PINT‐binding site (miR‐543‐Bio‐mut). E, miR‐543 expression in lung cancer tissues. F, miR‐543 expression in lung cancer cells (A549, H460, H1299, H1650) and WI‐38, HEL‐1 cells. G, miR‐543 expression in A549 and H1299 cells. Cells were infected with LV‐NC or LV‐LINC‐PINT for 48 h. Three independent experiments were carried out. Error bars stand for the mean ± SD of triplicate experiments. *P < .05

LncRNAs can sponge miRNAs and serve as competing endogenous RNAs in cancers. Here miR‐543 was predicted as a target of LINC‐PINT and their binding sites were exhibited in Figure 5A. Meanwhile, nine other microRNAs could also be predicted as the targets of LINC‐PINT as shown in Figure S1. Next, in Figure S2, we observed that LINC‐PINT was most enriched by miR‐543 in A549 cells compared to the other nine microRNAs. As displayed, miR‐543 mimics inhibited the WT‐LINC‐PINT luciferase activity in A549 cells (Figure 5B, *P* < .01). Next, RIP assay indicated LINC‐PINT and miR‐543 were more enriched in A549 cells (Figure 5C, *P* < .01). RNA‐pull down assay also confirmed the correlation between them (Figure 5D, *F* value = 256.7, *P* < .01). Moreover, in Figure 5E (*F* value = 24.06, *P* < .01) and 5F (*P* < .01), miR‐543 was greatly elevated in lung cancer. Overexpression of LINC‐PINT inhibited miR‐543 expression in lung cancer cells (Figure 5G). These data revealed miR‐543 was a direct target of LINC‐PINT.

### 3.6 PTEN was a direct target of MIR‐543

**Figure 6:**
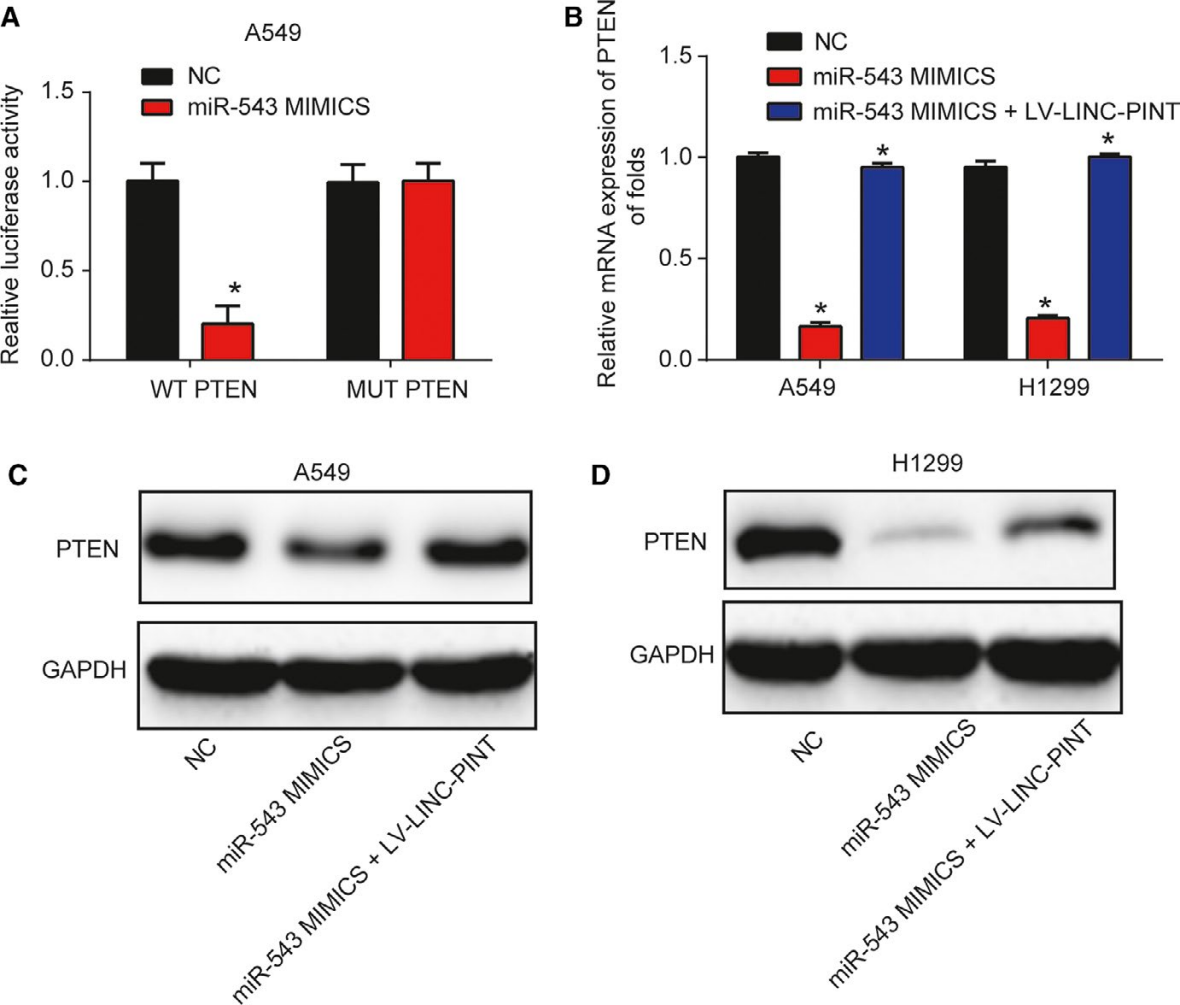
PTEN was a direct target of miR‐543. A, Luciferase activity was tested in A549 cells transfected with miR‐543 mimics, miR‐NC, WT‐PTEN, and MUT‐PTEN plasmid. B, PTEN mRNA expression in A549 and H1299 cells. Cells were infected with miR‐543 mimics for 48 h and then, infected with LV‐LINC‐PINT. C‐D, PTEN protein expression in A549 and H1299 cells. *P < .05

PTEN was predicted as the target of miR‐543. Here, we carried out the luciferase reporter assay and the result in Figure 6A (*P* < .01) demonstrated miR‐543 mimics reduced the WT‐PTEN luciferase activity rather than that of the MUT‐PTEN. In Figure 6B‐D (*P* < .01), we observed that miR‐543 mimics repressed PTEN expression, which was reversed by overexpression of LINC‐PINT. These data implied PTEN was a direct target of miR‐543.

### 3.7 LINC‐PINT inhibited lung cancer cell tumorigenicity in vivo

**Figure 7:**
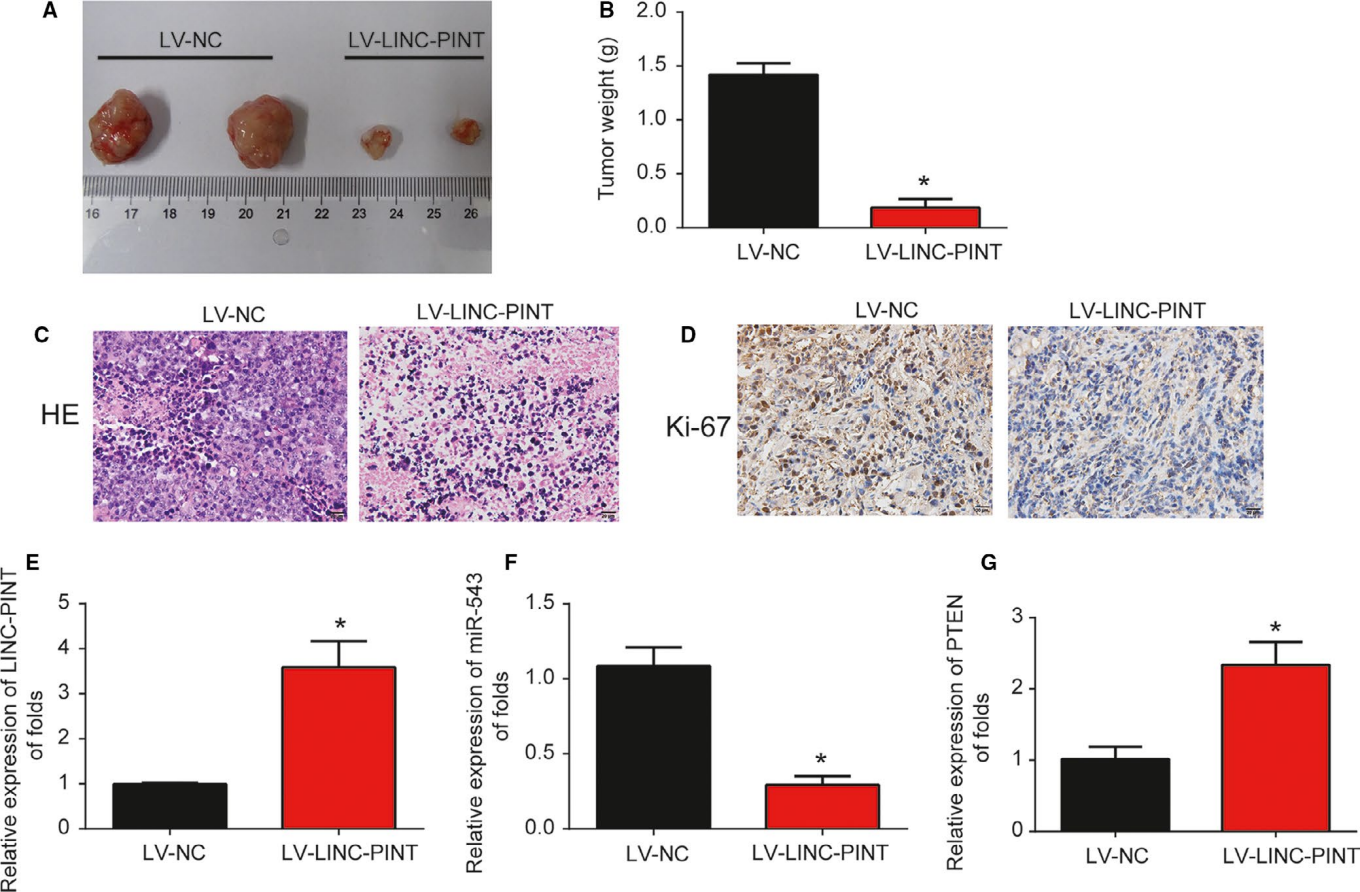
Overexpression of LINC‐PINT repressed lung cancer progression through regulating miR‐543 and PTEN in vivo. Twelve 8‐week‐old female BALB/c nude mice were injected with A549 cells infected with LV‐NC (six mice) or LV‐LINC‐PINA (six mice). (A) Solid tumors were picked from mouse subcutaneous tissue. (B) Tumor weight was determined after infection of LINC‐PINT or NC for 30 d. H&E staining (C) and IHC staining of Ki‐67 (D) in tumor tissues. The level of LINC‐PINT (E), miR‐543 (F), and PTEN (G) in xenograft tumors were analyzed by qRT‐PCR. Three independent experiments were carried out. Error bars stand for the mean ± SD triplicate experiments. *P < .05

After studying the in vitro roles of LINC‐PINT, in vivo assays were conducted in a xenograft tumor model. A549 cells were infected with LV‐NC or LV‐LINC‐PINT. In Figure 7A,B (*P* < .01), the tumors in the two groups were peeled and we found that LV‐LINC‐PINT greatly inhibited the tumor volume and tumor weight. HE staining and the IHC exhibited Ki‐67 was significantly repressed by LINC‐PINT (Figure 7C,D, *P* < .01). Finally, qRT‐PCR data revealed that LINC‐PINT was upregulated, miR‐543 was reduced, while PTEN was induced in the LV‐LINC‐PINT group (Figure 7E‐G, *P* < .01). These suggested that LINC‐PINT inhibited lung cancer progression via regulating miR‐543 and PTEN in vivo.

## 4 DISCUSSION

Accumulating biological roles of lncRNAs have been reported in lung cancer progression.^18^, ^19^, ^20^ For example, MALAT1 serves as an important regulator of lung cancer.^21^ Knockdown of PVT1 can enhance NSCLC radiosensitivity by sponging miR‐195.^22^ In addition, XIST promotes NSCLC progression by modulating miR‐186‐5p.^23^ In recent years, a great deal of evidence has proved that LINC‐PINT can exert an important role in tumors. For example, downregulated LINC‐PINT has been demonstrated to be correlated with the advanced tumor stage and bad survival in various cancers.

Here, we reported LINC‐PINT was greatly reduced whereas miR‐543 was upregulated in lung cancer. Overexpression of LINC‐PINT greatly inhibited A549 and H1299 cell proliferation, colony formation, induced the apoptosis, blocked cell cycle and restrained cell migration/invasion ability. miR‐543 acted as a downstream target of LINC‐PINT and PTEN was a potential target of miR‐543. These data were basically consistent with those previous studies and it was indicated that LINC‐PINT functioned as a tumor inhibitor in lung cancer. LINC‐PINT is involved in various cancers, such as gastric cancer, glioblastoma, and acute lymphoblastic leukemia. For example, In gastric cancer, LINC‐PINT is decreased and it can predict the poor survival.^24^ In glioblastoma, a peptide, which is encoded by LINC‐PINT represses the oncogenic transcriptional elongation.^25^ In acute lymphoblastic leukemia, deregulation of LINC‐PINT participates in the abnormal proliferation of leukemic cells.^26^ In addition, LINC‐PINT can function as a tumor inhibitor through sponging miR‐543 and miR‐576‐5p in esophageal cancer.^27^ In lung cancer, LINC‐PINT can repress NSCLC progression via sponging miR‐218‐5p and PDCD4.^28^ Here, we measured LINC‐PINT expression in lung cancer tissues and cells. LINC‐PINT was decreased in NSCLC. Meanwhile, LINC‐PINT greatly repressed lung cancer progression in vitro. Additionally, our data revealed LINC‐PINT could depress tumor growth in vivo.

miR‐543 was predicated as the target of LINC‐PINT. miR‐543 is located on human chromosome 14.^29^ miR‐543 is dysregulated in many cancers, such as hepatocellular carcinoma, colorectal cancer, and glioma.^30^, ^31^, ^32^ This study confirmed miR‐543 acted as a target of LINC‐PINT. miR‐543 was significantly increased in lung cancer. Moreover, miR‐543 was negatively regulated by LINC‐PINT.

Furthermore, we found that PTEN was a target of miR‐543. The association between them was validated in our study. miR‐543 restrained PTEN expression and LINC‐PINT rescued that in lung cancer cells. More importantly, we found that LINC‐PINT could positively regulate PTEN expression by sponging miR‐543 in vitro and in vivo.

In conclusion, we reported that LINC‐PINT affected proliferation, metastasis and invasion in lung cancer via regulating miR‐543 and inducing PTEN. In sum, our study exhibited that LINC‐PINT functioned as a tumor inhibitor during NSCLC progression and revealed a novel ceRNA regulatory pathway by sponging miR‐543 and activating PTEN. Thus, LINC‐PINT was potentially a novel therapeutic target for lung cancer patients. These results implied that LINC‐PINT might become an effective target for lung cancer.

## CONFLICT OF INTEREST

The authors declare that they have no conflict of interest.

## AUTHOR CONTRIBUTION

XC, NL, and SW designed the concept and experiments; SW, WJ, XZ, and ZL performed the experiments; QG and WW collected the data and did the analysis. SW and NL prepared the manuscript draft. XC revised the manuscript. All the authors approved the final proof.

## Data Availability

The datasets used during this study are available from the corresponding author on request.

## References

[1] Tsao AS , Scagliotti GV , Bunn PA , et al. Scientific advances in lung cancer 2015. J Thoracic Oncol. 2016;11(5):613‐638. 10.1016/j.jtho.2016.03.012 27013409

[2] Wistuba II . Genetics of preneoplasia: lessons from lung cancer. Curr Mol Med. 2007;7(1):3‐14. 17311529 10.2174/156652407779940468

[3] Zheng M . Classification and pathology of lung cancer. Surg Oncol Clin N Am. 2016;25(3):447‐468. 27261908 10.1016/j.soc.2016.02.003

[4] Bansal P , Osman D , Gan GN , Simon GR , Boumber Y . Recent advances in targetable therapeutics in metastatic non‐squamous NSCLC. Front Oncol. 2016;6:112. 27200298 10.3389/fonc.2016.00112 PMC4854869

[5] Mercer TR , Dinger ME , Mattick JS . Long non‐coding RNAs: insights into functions. Nat Rev Genet. 2009;10(3):155‐159. 19188922 10.1038/nrg2521

[6] Tsai MC , Spitale RC , Chang HY . Long intergenic noncoding RNAs: new links in cancer progression. Can Res. 2011;71(1):3‐7. 10.1158/0008-5472.CAN-10-2483 PMC3057914 21199792

[7] Gutschner T , Diederichs S . The hallmarks of cancer: a long non‐coding RNA point of view. RNA Biol. 2012;9(6):703‐719. 22664915 10.4161/rna.20481 PMC3495743

[8] Bhan A , Soleimani M , Mandal SS . Long noncoding RNA and cancer: a new paradigm. Can Res. 2017;77(15):3965‐3981. 10.1158/0008-5472.CAN-16-2634 PMC8330958 28701486

[9] Gao P , Wei GH . Genomic insight into the role of lncRNA in cancer susceptibility. Int J Mol Sci. 2017;18(6):e1239. 28598379 10.3390/ijms18061239 PMC5486062

[10] Wei GH , Wang X . lncRNA MEG3 inhibit proliferation and metastasis of gastric cancer via p53 signaling pathway. Eur Rev Med Pharmacol Sci. 2017;21(17):3850‐3856. 28975980

[11] Zhang W , Yuan W , Song J , Wang S , Gu X . LncRna CPS1‐IT1 suppresses cell proliferation, invasion and metastasis in colorectal cancer. Cellular Physiol Biochem. 2017;44(2):567‐580. 29145177 10.1159/000485091

[12] Cui Y , Zhang F , Zhu C , Geng L , Tian T , Liu H . Upregulated lncRNA SNHG1 contributes to progression of non‐small cell lung cancer through inhibition of miR‐101‐3p and activation of Wnt/beta‐catenin signaling pathway. Oncotarget. 2017;8(11):17785‐17794. 28147312 10.18632/oncotarget.14854 PMC5392286

[13] Jia X , Wang Z , Qiu L , et al. Upregulation of LncRNA‐HIT promotes migration and invasion of non‐small cell lung cancer cells by association with ZEB1. Cancer Med. 2016;5(12):3555‐3563. 27790864 10.1002/cam4.948 PMC5224854

[14] Li LE , Zhang G‐Q , Chen H , et al. Plasma and tumor levels of Linc‐pint are diagnostic and prognostic biomarkers for pancreatic cancer. Oncotarget. 2016;7(44):71773‐71781. 27708234 10.18632/oncotarget.12365 PMC5342121

[15] Marín‐Béjar O , Mas AM , González J , et al. The human lncRNA LINC‐PINT inhibits tumor cell invasion through a highly conserved sequence element. Genome Biol. 2017;18(1):202. 29078818 10.1186/s13059-017-1331-y PMC5660458

[16] Lu H , Yang D , Zhang L , et al. Linc‐pint inhibits early stage pancreatic ductal adenocarcinoma growth through TGF‐beta pathway activation. Oncol Lett. 2019;17(5):4633‐4639. 30944652 10.3892/ol.2019.10111 PMC6444384

[17] Zhu J , Gu H , Lv X , Yuan C , Ni P , Liu F . LINC‐PINT activates the mitogen‐activated protein kinase pathway to promote acute myocardial infarction by regulating miR‐208a‐3p. Circulation J. 2018;82(11):2783‐2792. 10.1253/circj.CJ-18-0396 30249926

[18] Wei MM , Zhou GB . Long non‐coding RNAs and their roles in non‐small‐cell lung Cancer. Genomics, Proteomics Bioinformatics. 2016;14(5):280‐288. 27397102 10.1016/j.gpb.2016.03.007 PMC5093404

[19] Chen Y , Li C , Pan Y , et al. The emerging role and promise of long noncoding RNAs in lung cancer treatment. Cellular Physiol Biochem. 2016;38(6):2194‐2206. 27183839 10.1159/000445575

[20] Tao H , Yang JJ , Zhou X , Deng ZY , Shi KH , Li J . Emerging role of long noncoding RNAs in lung cancer: current status and future prospects. Respir Med. 2016;110:12‐19. 26603340 10.1016/j.rmed.2015.10.006

[21] Gutschner T , Hammerle M , Eissmann M , et al. The noncoding RNA MALAT1 is a critical regulator of the metastasis phenotype of lung cancer cells. Can Res. 2013;73(3):1180‐1189. 10.1158/0008-5472.CAN-12-2850 PMC3589741 23243023

[22] Wu D , Li Y , Zhang H , Hu X . Knockdown of Lncrna PVT1 enhances radiosensitivity in non‐small cell lung cancer by sponging Mir‐195. Cellular Physiol Biochem. 2017;42(6):2453‐2466. 28848163 10.1159/000480209

[23] Wang H , Shen Q , Zhang X , et al. The long non‐coding RNA XIST controls non‐small cell lung cancer proliferation and invasion by modulating miR‐186‐5p. Cellular Physiol Biochem. 2017;41(6):2221‐2229. 28448993 10.1159/000475637

[24] Feng H , Zhang J , Shi Y , Wang L , Zhang C , Wu L . Long noncoding RNA LINC‐PINT is inhibited in gastric cancer and predicts poor survival. J Cell Biochem. 2019;120(6):9594‐9600. 30569513 10.1002/jcb.28236

[25] Zhang M , Zhao K , Xu X , et al. A peptide encoded by circular form of LINC‐PINT suppresses oncogenic transcriptional elongation in glioblastoma. Nat Commun. 2018;9(1):4475. 30367041 10.1038/s41467-018-06862-2 PMC6203777

[26] Garitano‐Trojaola A , Jose‐Eneriz ES , Ezponda T , et al. Deregulation of linc‐PINT in acute lymphoblastic leukemia is implicated in abnormal proliferation of leukemic cells. Oncotarget. 2018;9(16):12842‐12852. 29560114 10.18632/oncotarget.24401 PMC5849178

[27] Zhang L , Chen J , Wang L , et al. Linc‐PINT acted as a tumor suppressor by sponging miR‐543 and miR‐576‐5p in esophageal cancer. J Cell Biochem. 2019;120(12):19345‐19357. 31464068 10.1002/jcb.28699

[28] Zhang L , Hu J , Li J , Yang Q , Hao M , Bu L . Long noncoding RNA LINC‐PINT inhibits non‐small cell lung cancer progression through sponging miR‐218‐5p/PDCD4. Artificial Cells, Nanomed Biotechnol. 2019;47(1):1595‐1602. 10.1080/21691401.2019.1605371 31010333

[29] Haga CL , Phinney DG . MicroRNAs in the imprinted DLK1‐DIO3 region repress the epithelial‐to‐mesenchymal transition by targeting the TWIST1 protein signaling network. J Biol Chem. 2012;287(51):42695‐42707. 23105110 10.1074/jbc.M112.387761 PMC3522270

[30] Yu L , Zhou L , Cheng Y , et al. MicroRNA‐543 acts as an oncogene by targeting PAQR3 in hepatocellular carcinoma. Am J Cancer Res. 2014;4(6):897‐906. 25520877 PMC4266721

[31] Fan C , Lin Y , Mao Y , et al. MicroRNA‐543 suppresses colorectal cancer growth and metastasis by targeting KRAS, MTA1 and HMGA2. Oncotarget. 2016;7(16):21825‐21839. 26968810 10.18632/oncotarget.7989 PMC5008326

[32] Xu L , Yu J , Wang Z , Zhu Q , Wang W , Lan Q . miR‐543 functions as a tumor suppressor in glioma in vitro and in vivo. Oncol Rep. 2017;38(2):725‐734. 28627653 10.3892/or.2017.5712 PMC5562083

