# Supplementary figures and images for: LINC‐PINT alleviates lung cancer progression via sponging miR‐543 and inducing PTEN
Source: Cancer Med. 2020 Jan 25;9(6):1999–2009. doi: 10.1002/cam4.2822 (PMC7064031; doi:10.1002/cam4.2822)

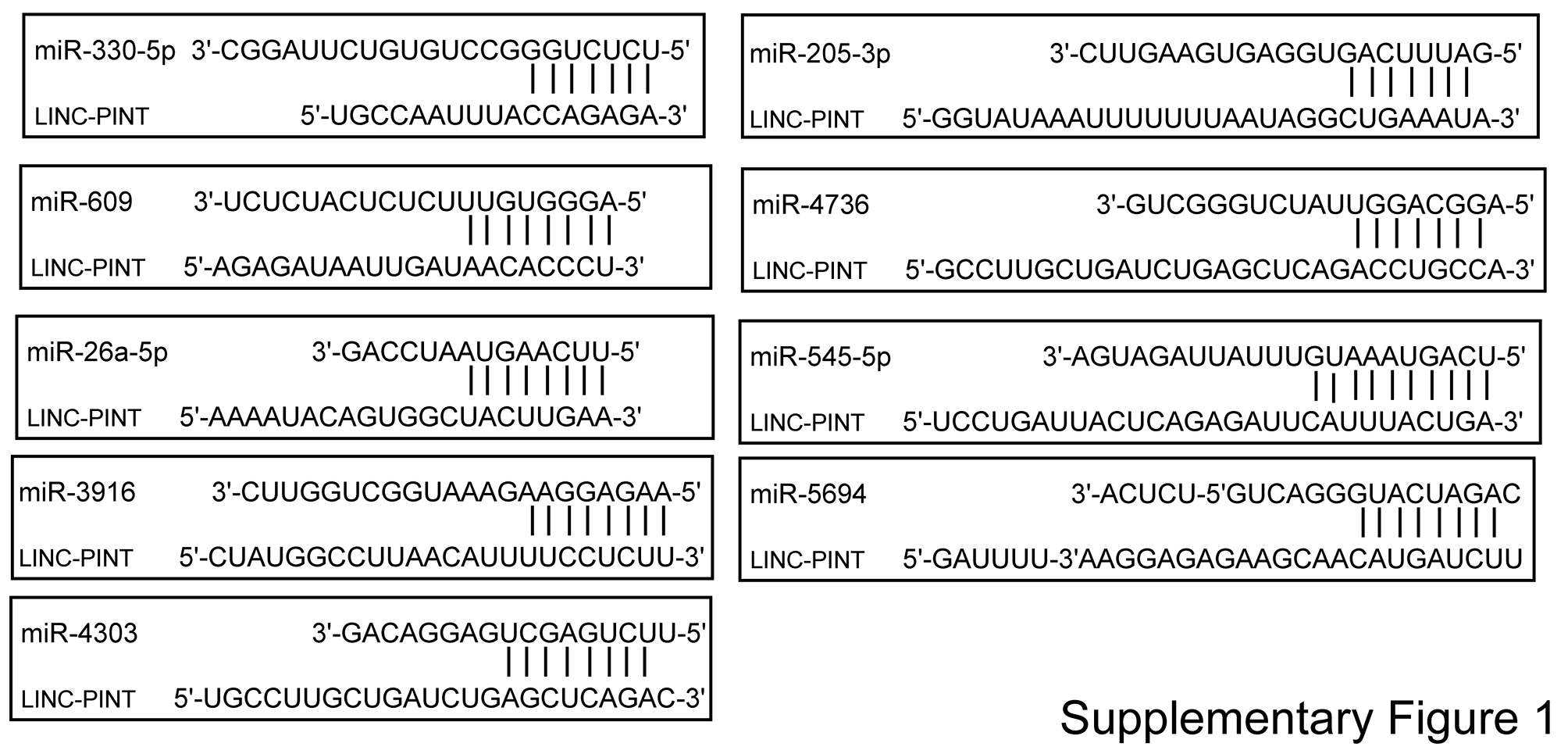

Supplement: Supplementary file 1 [file CAM4-9-1999-s001.tif]

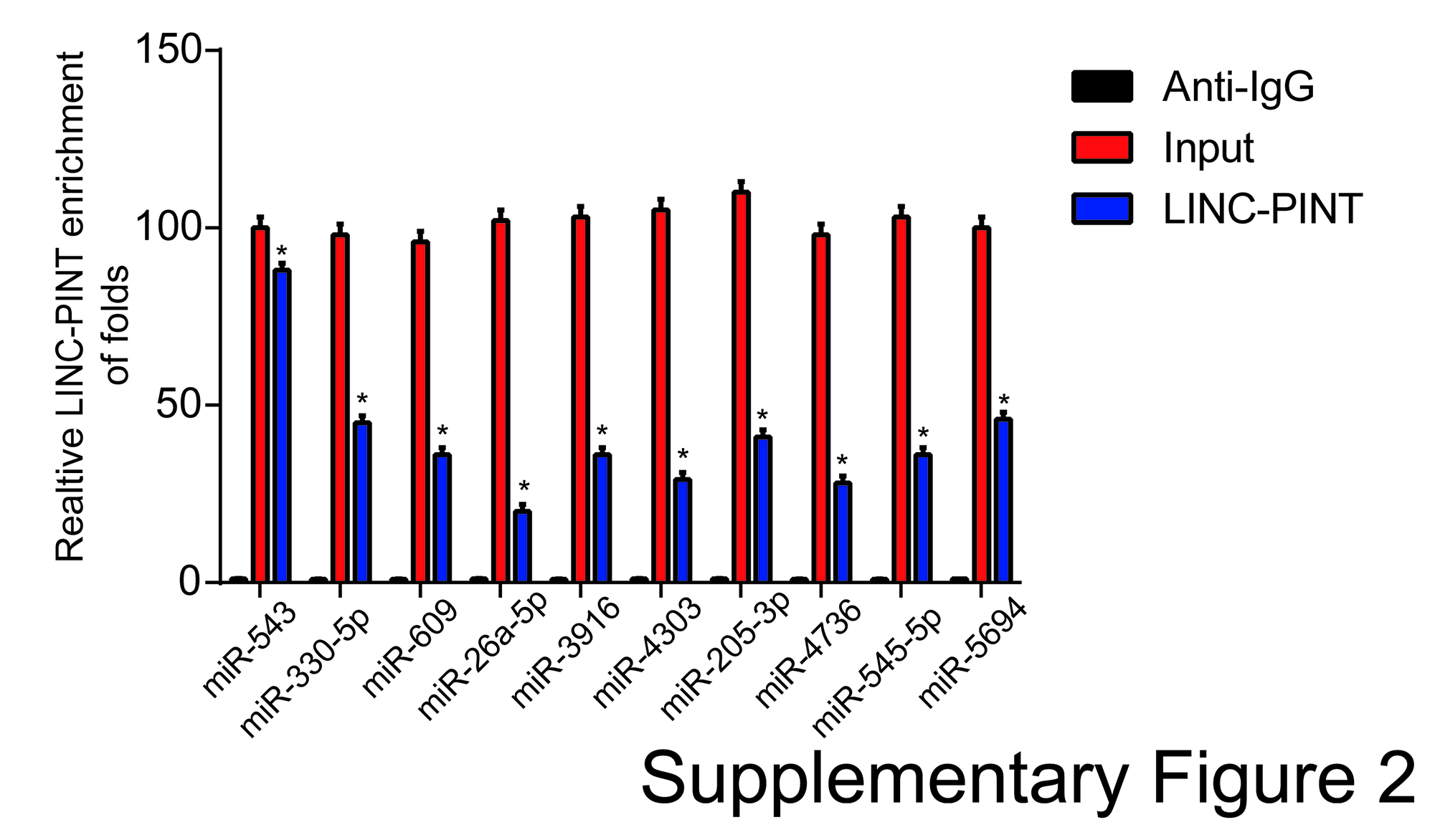

Supplement: Supplementary file 2 [file CAM4-9-1999-s002.tif]
