# Supplementary material for: LINC‐PINT alleviates lung cancer progression via sponging miR‐543 and inducing PTEN
Source: Cancer Med. 2020 Jan 25;9(6):1999–2009. doi: 10.1002/cam4.2822 (PMC7064031; doi:10.1002/cam4.2822)
Supplement: Supplementary file 3 [file CAM4-9-1999-s003.docx]

**Supplementary figure legends**

**Supplementary Figure 1:** Binding sites between LINC-PINT and miR-330-5p, miR-609, miR-26a-5p, miR-3916, miR-4303, miR-205-3p, miR-4736, miR-545-5p，miR-5694.

**Supplementary Figure 2:** RIP assay implied that LINC-PINT were most enriched by miR-543 in A549 cells compared to the other nine microRNAs.
